# Mapping cortical haemodynamics during neonatal seizures using diffuse optical tomography: A case study

**DOI:** 10.1016/j.nicl.2014.06.012

**Published:** 2014-07-06

**Authors:** Harsimrat Singh, Robert J. Cooper, Chuen Wai Lee, Laura Dempsey, Andrea Edwards, Sabrina Brigadoi, Dimitrios Airantzis, Nick Everdell, Andrew Michell, David Holder, Jeremy C. Hebden, Topun Austin

**Affiliations:** a neoLAB, The Evelyn Perinatal Imaging Centre, Rosie Hospital, Cambridge CB2 0QQ, UK; b Department of Medical Physics and Bioengineering, University College London, London WC1E 6BT, UK; c Neonatal Unit, Rosie Hospital, Cambridge University Hospitals NHS Foundation Trust, Cambridge CB2 0QQ, UK; d Department of Developmental Psychology, University of Padova, Padova, Italy; e Department of Neurophysiology, Addenbrooke's Hospital, Cambridge University Hospitals NHS Foundation Trust, Cambridge CB2 0QQ, UK

**Keywords:** Diffuse optical tomography (DOT), Neonatal seizures, Functional near infrared spectroscopy (fNIRS), Hypoxic–ischaemic encephalopathy (HIE)

## Abstract

Seizures in the newborn brain represent a major challenge to neonatal medicine. Neonatal seizures are poorly classified, under-diagnosed, difficult to treat and are associated with poor neurodevelopmental outcome. Video-EEG is the current gold-standard approach for seizure detection and monitoring. Interpreting neonatal EEG requires expertise and the impact of seizures on the developing brain remains poorly understood. In this case study we present the first ever images of the haemodynamic impact of seizures on the human infant brain, obtained using simultaneous diffuse optical tomography (DOT) and video-EEG with whole-scalp coverage. Seven discrete periods of ictal electrographic activity were observed during a 60 minute recording of an infant with hypoxic–ischaemic encephalopathy. The resulting DOT images show a remarkably consistent, high-amplitude, biphasic pattern of changes in cortical blood volume and oxygenation in response to each electrographic event. While there is spatial variation across the cortex, the dominant haemodynamic response to seizure activity consists of an initial increase in cortical blood volume prior to a large and extended decrease typically lasting several minutes. This case study demonstrates the wealth of physiologically and clinically relevant information that DOT–EEG techniques can yield. The consistency and scale of the haemodynamic responses observed here also suggest that DOT–EEG has the potential to provide improved detection of neonatal seizures.

## Highlights

• We present the first DOT images of seizures in an infant with whole-scalp coverage.
• In this infant, seizures produce consistent, large, biphasic haemodynamic responses.
• DOT reveals spatial variation in seizure response despite generalised EEG activity.

## 1 Introduction

Neonatal brain injury is a significant cause of lifelong disability. Seizures are a common symptom of brain injury in the newborn infant, but they are poorly classified, frequently under-diagnosed, and are difficult to treat (Rennie and Boylan, 2007; van Rooij et al., 2013a,b). They are also independently associated with poor neurodevelopmental outcome (Payne et al., 2014; van Rooij et al., 2013a). Monitoring with video-synchronized electroencephalography (video-EEG) has become the clinical gold standard for identifying seizures (Connell et al., 1987, 1989; Hahn and Riviello, 2004; Smith, 2005; Shellhaas et al., 2011). However, application and interpretation of neonatal EEG are challenging and require expertise. It is therefore rarely used for continuous monitoring. As a result, seizures are commonly identified through clinical observation, despite the fact that clinical manifestations can be extremely subtle or absent in the newborn (Pinto and Giliberti, 2001; Weiner et al., 1991). In some cases clinical manifestations of seizures are apparent despite the EEG being seizure-negative (van Rooij et al., 2010); frontal seizures, for example, often present no scalp EEG correlate. This observation is unsurprising given the complex relationship between neuronal hyperactivity, neuronal synchrony and the EEG signal that is observed at the scalp. Direct comparisons between intracranial and scalp EEG approaches have shown that epileptiform discharges occurring in the cortex are not always apparent on the scalp (Ray et al., 2007; Tao et al., 2005).

Localized changes in haemodynamic parameters such as the concentration of oxy- and deoxy-haemoglobin (HbO and HbR respectively) have long been used as proxy measures for changes in neuronal activity (Boas et al., 2014; Obrig and Villringer, 2003). In the healthy brain, the changes in local haemodynamic parameters are tightly coupled to the metabolic demand of surrounding neurons. Whether this relationship is maintained during seizures is still a matter of debate, and has been investigated extensively in adults, in children and in animal models using functional magnetic resonance imaging (fMRI), positron emission tomography (PET) and optical approaches (Avery et al., 2000; Suh et al., 2005; Zhao et al., 2007; Zimmermann et al., 2008; Chaudhary et al., 2013; Zhao et al., 2011). Infant studies are more challenging due to the unpredictable nature of seizures and the vulnerability of the cohort, but there is growing evidence that seizures in the infant are not just a symptom of brain injury, but can cause injury themselves (Legido et al., 1991; McBride et al., 2000; van Rooij et al., 2010; West et al., 2011).

Near-infrared spectroscopy (NIRS) has been used to investigate the infant brain for over 25 years (Lloyd-Fox et al., 2010; Boas et al., 2014). This technique is non-invasive, low-cost and can be readily employed at the cot-side in the intensive care environment without interfering with other physiological recording systems (including EEG, which may be recorded simultaneously). Recent advances in near-infrared hardware and image reconstruction methods have made it possible to produce three-dimensional images of changes in haemodynamic parameters in the brain. Such techniques, which use multiple sources and detectors of near-infrared light, are commonly referred to as diffuse optical tomography (DOT) (Joseph et al., 2006; Arridge, 2011; Zeff et al., 2007; Eggebrecht et al., 2012).

Wallois et al. (2009, 2010) were the first to observe the haemodynamic response to neonatal seizures in humans. Haemodynamic features were isolated using single-channel NIRS during the onset of ‘seizure-like’ EEG discharges in an infant. This response consisted of an increase in HbO and HbR, followed by an undershoot of HbR only, and a slow return to baseline. A previous study by our group (Cooper et al., 2012) used DOT–EEG to reveal the existence of transient haemodynamic events in neonates who had previously experienced electrographic seizures. These events consisted of an increase in HbO and HbR followed by a rapid decrease in the concentration of both parameters with slow recovery back to baseline. These biphasic features were of untypically high amplitude and lasted up to 2 min. The EEG traces recorded simultaneously did not exhibit any distinct abnormality during the observed haemodynamic events. It was therefore not possible to associate these events with electrographic seizures, but they were not observed in a large number of healthy, age-matched controls.

Intrinsic optical imaging with simultaneous EEG has been used in adult studies to investigate seizures in the exposed cortex intra-operatively (Zhao et al., 2007). A focal increase in both blood volume and HbR was observed during seizure activity, indicating that perfusion was not adequate to meet localized metabolic demand. Remarkably, the observed haemodynamic changes preceded the onset of the electrographic seizures by approximately 20 s, a phenomenon that has been observed repeatedly (Zhao et al., 2011; Ma et al., 2013).

Because the spatial and temporal relationship between ictal electrical activity and cerebral haemodynamics remains unclear, particularly in the newborn, we have begun a series of studies of neonatal seizures using diffuse optical tomography and EEG with whole-scalp coverage. These studies aim to improve our understanding of the haemodynamic variations associated with neonatal seizures and neonatal neuropathology in general. Specifically, we seek to determine a) whether neonatal seizures produce a measurable and consistent DOT signal, b) whether this DOT signal can provide a clearer indication of seizure events than EEG alone and c) whether seizure-induced haemodynamic changes provide a mechanism to exacerbate brain injury.

In this paper, we present images which are, to our knowledge, the first ever DOT images recorded in an infant with seizures. The simultaneously recorded video-EEG allows us to investigate the temporal and spatial relationship between electrographic seizures and the haemodynamic signal across the cortex.

## 2 Materials and methods

### 2.1 Patient history

The patient was a female infant born by spontaneous vaginal delivery at 40 weeks and 4 d postmenstrual age. The infant had an abnormal cardiotocography (CTG) and at birth presented without heart rate or respiratory effort. Full resuscitation was commenced and the infant was admitted with severe hypoxic–ischaemic encephalopathy to the neonatal intensive care unit of the Rosie Hospital. A 72-hour period of therapeutic hypothermia (with a target core temperature of 33.5 °C) was initiated shortly after birth, followed by a re-warming period where the core temperature was increased at the rate of 0.5 °C per hour until the infant reached a normal temperature. Therapeutic hypothermia provides a neuroprotective effect and is standard clinical practice in term infants who have suffered a hypoxic–ischaemic insult.

Amplitude-integrated EEG via a cerebral function monitor (CFM) (Olympic CFM™ 6000, Natus Medical Inc., California) recorded seizures during both the cooling and re-warming phases with evidence of clinical manifestation despite treatment with anticonvulsants including phenobarbital, clonazepam and phenytoin. A structural magnetic resonance imaging (MRI) scan on day 7 showed abnormal signal intensity in the basal ganglia, brainstem and corpus callosum. It also indicated thromboses in major sinuses and dilation of medullary intra-cerebral veins and internal cerebral veins. Informed written parental consent was obtained for a simultaneous DOT–EEG recording, approved by the National Research Ethics Service Committee East of England (REC reference 09/H0308/125).

### 2.2 Data acquisition

**Fig. 1:**
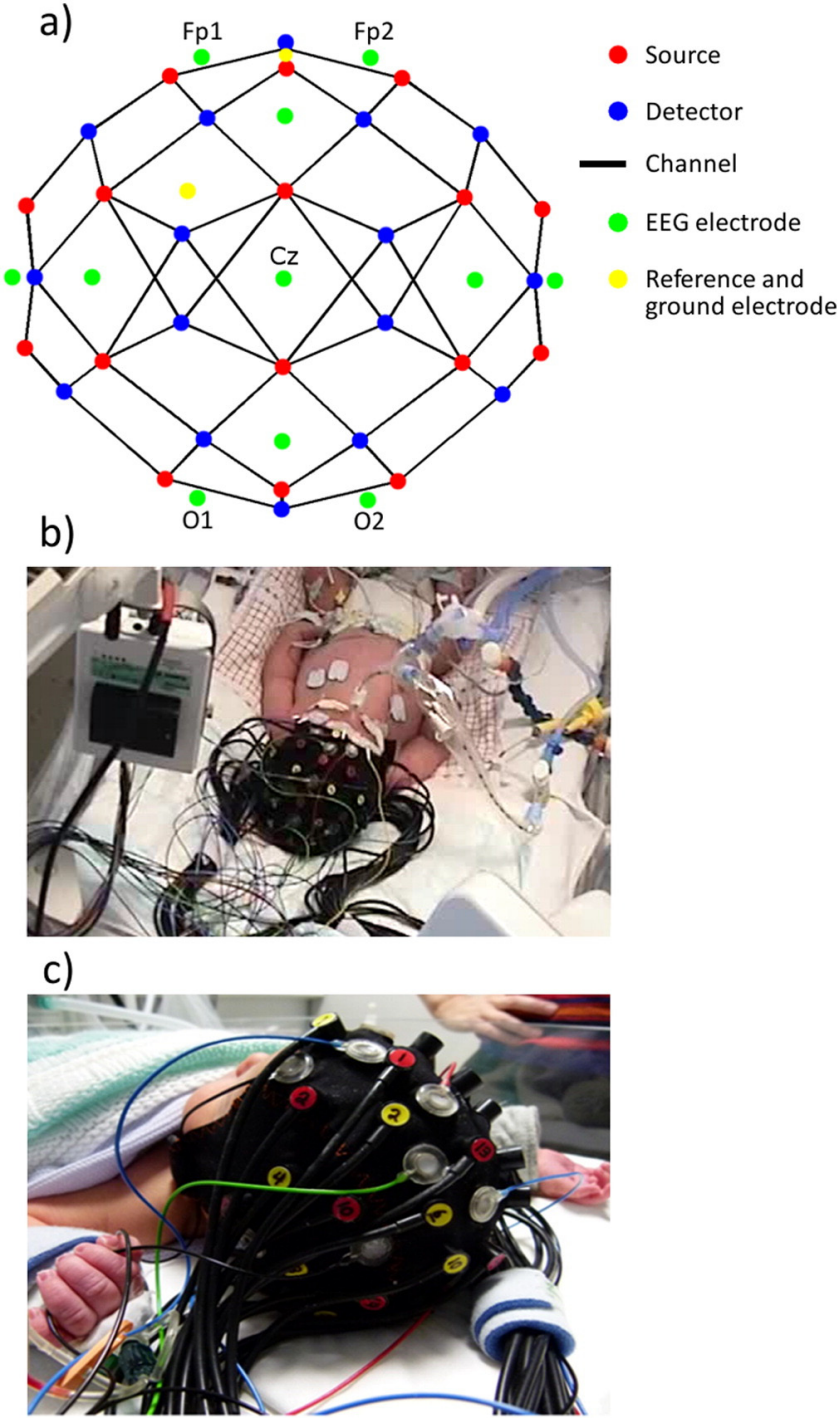
Panel a) shows a two-dimensional representation of the array design including the positions of the sources, detectors and the EEG electrodes. Panel b) provides an example frame from the video-EEG and panel c) shows a photograph of the DOT–EEG array on an infant's head.

Simultaneous DOT–EEG was performed over a 60 minute period after the re-warming phase, during which the infant showed no signs of consciousness and exhibited little motion. DOT data were acquired using the UCL Diffuse Optical Tomography System (Everdell et al., 2005), a continuous-wave system consisting of 16 dual wavelength laser diode sources illuminating at 780 and 850 nm and 16 avalanche photodiode detectors (APDs). Frequency multiplexing allows all sources to be illuminated simultaneously. The system has a sampling rate of 10 Hz. The sources and detectors were coupled to the infant's head using optical fibre bundles attached to a soft, flexible head cap (EasyCap, Germany). The cap has a circumference of 34 cm, matched to the age and size of the infant. The optical fibres were positioned within the cap at a subset of locations taken from the 10–5 system (Oostenveld and Praamstra, 2001). These locations were chosen to maximize the density of DOT source–detector pairs (channels) with optimal separations between 20 and 40 mm while also covering the whole scalp. The array design, which provides a total of 58 dual-wavelength DOT channels, is shown in Fig. 1.

Eleven annular Ag/AgCl electrodes (EasyCap Gmbh, Germany) were also coupled directly to the cap in a standard neonatal EEG montage (Fp1, Fp2, Fz, T3, T4, C3, Cz, C4, Pz, O1, O2) with reference and ground electrodes at AFpz and FC1 respectively. Video-EEG recordings were performed using a Micromed SystemPlus clinical EEG system (Micromed S.p.A., Italy) with a sampling frequency of 256 Hz. The EEG data were band-pass filtered (0.3–70 Hz) and notch filtered at 50 Hz to eliminate main interference. A single channel electrocardiogram (ECG) was recorded using the auxiliary channels of the EEG system.

In order to synchronize the DOT and EEG data, a custom-built external pulse generator was programmed to send a predetermined pattern of electrical pulses to both systems during recording. The pulse events within the two datasets could then be aligned during data processing and analysis.

## 3 Data analysis

### 3.1 DOT data pre-processing

DOT data were initially inspected using the HOMER2 toolbox (http://homer-fnirs.org; Huppert et al., 2009). Channels which exhibited very low optical intensities were excluded. A software error caused a discontinuity in the measured intensities of 5 channels at approximately 1080 s into the recording. This artifact was corrected using a spline interpolation approach developed for motion artifact correction (Scholkmann et al., 2010; Cooper et al., 2012). The data were low-pass filtered with a cut-off of 1 Hz to remove heart rate and high frequency noise. The intensity values for each wavelength and channel were converted to changes in optical density relative to the mean of each channel across the entire acquisition period. For the channel-wise analysis of data, the changes in optical density were converted to changes in concentration of oxy-haemoglobin, deoxy-haemoglobin, and total haemoglobin (or HbT = HbO + HbR) using the modified Beer–Lambert law with an estimated differential pathlength factor of 4.9 (Scholkmann and Wolf, 2013). A channel-wise mean haemodynamic signal was obtained in order to help identify the dominant features of the haemodynamic data.

### 3.2 EEG and video processing

The EEG was reviewed in standard longitudinal and transverse bipolar montages as well as average reference montage, commonly employed in clinical neonatal EEG. Clinical inspection and reporting of the video-EEG data was undertaken independently by two clinical neurophysiologists (AM and DH) to identify the onset and duration of any seizures. Neither neurophysiologist had access to the diffuse optical data prior to their inspection of the video-EEG.

Further EEG analysis was performed using the FIELDTRIP toolbox (Oostenveld et al., 2011). A simple paired *t*-test was used to examine the statistical difference between the EEG power recorded over the left and right hemispheres.

The time-locked video was acquired at 25 frames per second (fps) with a resolution of 720 × 576 pixels. This was down-sampled to 5 fps with a resolution of 300 × 220 pixels. The video data was then synchronized to the processed EEG and DOT datasets for comparison of the time-courses.

### 3.3 MRI-informed optical head modelling and image reconstruction

A series of structural MRI sequences of the patient were obtained as part of the clinical care. These included axial and sagittal T1-weighted contrast, axial T2-weighted contrast and Spoiled Gradient Echo (SPGR) sequences. Despite the existence of multiple structural images, their large slice thickness and irregular voxel size (typically 1 × 1 × 4 mm) made accurate segmentation and finite element meshing of the patient's MRI extremely challenging. In order to utilize the available information present in the MRI while also producing finite element meshes free of large voxel errors, we employed the 4D neonatal optical head model package (Brigadoi et al., 2014) (http://www.ucl.ac.uk/medphys/research/4dneonatalmodel). This package contains a series of voxelized tissue masks and finite element meshes for the average neonatal head ranging from 29 to 44 weeks gestational age. This model was produced using the MRI atlas described by (Kuklisova-Murgasova et al., 2011). The age-matched voxelized tissue mask (which specifies extra-cerebral tissues, cerebrospinal fluid, grey matter and white matter tissue types) was spatially registered to an up-sampled version of the SPGR MRI image (Manjón et al., 2010) using ANTs (http://stnava.github.io/ANTs/; Avants et al., 2011) to produce a registered, 4-layer tissue mask with dimensions 256 × 256 × 132. The Iso2mesh package (http://iso2mesh.sourceforge.net; Fang and Boas, 2009) was used to take this registered volume and produce a high-density, multi-layered tetrahedral head mesh and a grey matter surface mesh.

The locations of structural landmarks of the infant's head (inion, nasion, etc.) were identified using the SPGR images. These landmarks allowed the 10–5 locations on the registered head mesh (and therefore the approximate optical fibre locations) to be determined. A forward model was computed using the TOAST optical imaging and reconstruction package. The tissue optical properties were based on a linear fit to the values reported by Strangman et al. (2003), Bevilacqua et al. (1999) and Ferradal et al. (2014) and are provided in Supplementary Table 1. These values were originally reported for adults. To our knowledge no infant-specific tissue optical properties are available, but they are thought to be similar to those of the adult (Fukui et al., 2003).

The locations of structural landmarks of the infant's head (inion, nasion, etc.) were identified using the SPGR images. These landmarks allowed the 10–5 locations on the registered head mesh (and therefore the approximate optical fibre locations) to be determined. A forward model was computed using the TOAST optical imaging and reconstruction package (Schweiger M. and Arridge S.R., 2014). The tissue optical properties were based on a linear fit to the values reported by (Strangman et al., 2003, Bevilacqua et al., 1999 and Ferradal et al., 2014) and are provided in Supplementary Table 1. These values were originally reported for adults. To our knowledge no infant-specific tissue optical properties are available, but they are thought to be similar to those of the adult (Fukui et al., 2003).

Multispectral linear reconstruction was performed with the linear regularization parameter set to 1% of the maximum single value decomposition of the Jacobian matrix (Corlu et al., 2005). The multispectral approach allows direct reconstruction of images of HbO and HbR. Images were initially reconstructed in the tetrahedral volume mesh. Cortical values were then projected to the grey matter surface mesh such that the value assigned to each grey matter surface node was the average of all high-density tetrahedral mesh nodes within a radius of 3 mm.

## 4 Results

### 4.1 Video-EEG analysis

**Fig. 2:**
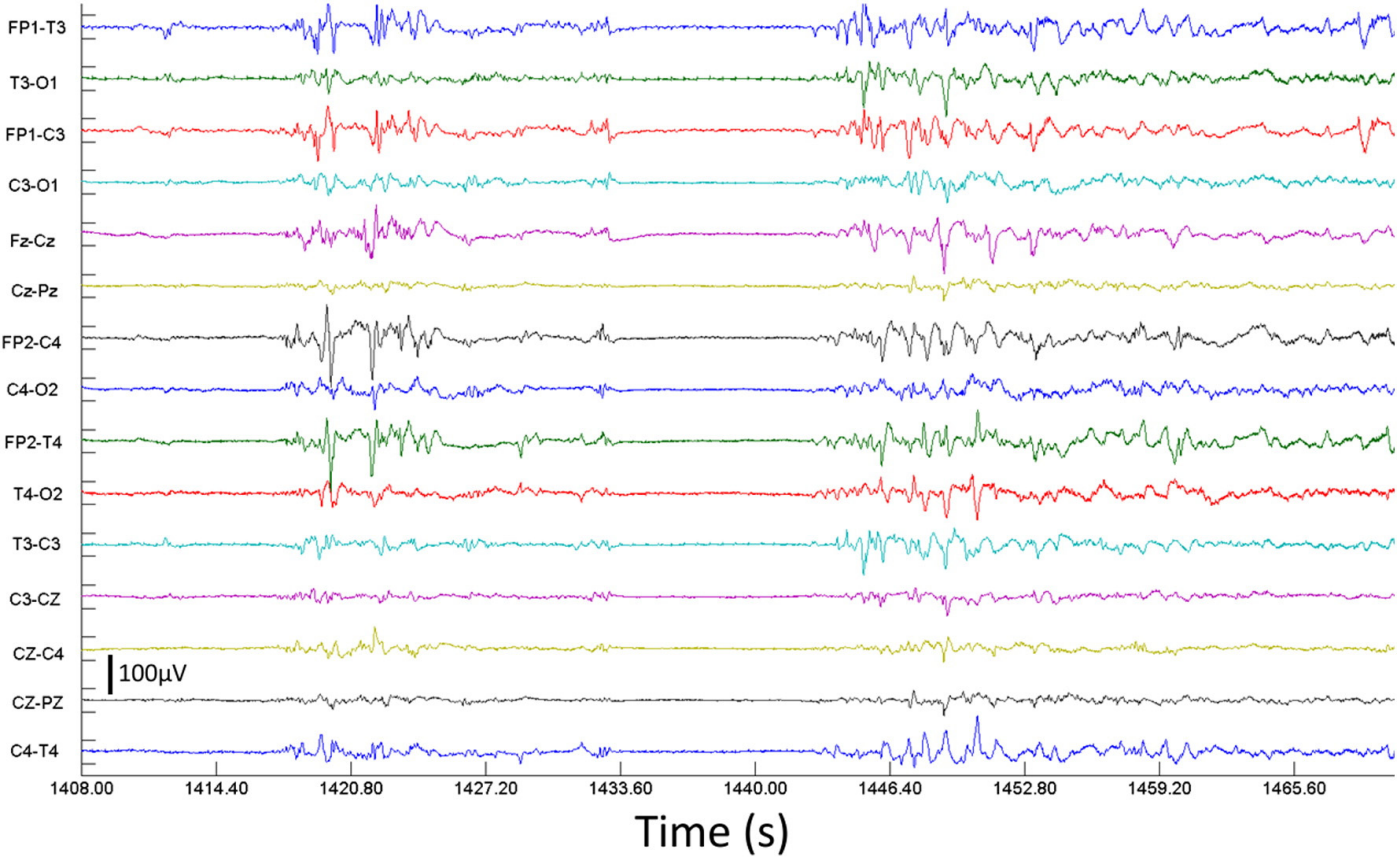
Bipolar EEG data showing the beginning of event 3 at 1442.5 s. A burst and a subsequent period of suppression are apparent in the 30 s leading up to the onset of the electrographic seizure.

Both neurophysiologists concluded that although clinical indications were not stereotyped for all episodes, the video presented some evidence of increased respiratory rate and abdominal jerking, possibly hiccups, which can be a manifestation of seizures. Seven distinct events were identified. Six of these were classified as seizures, and one (event 6) was briefer, with less well defined EEG evolution and clinical manifestations, considered a possible seizure. Ictal EEG showed sharpened high amplitude slowing at onset, including some 1 Hz slow activity. Superimposed 10–15 Hz activity was seen early in some events. These features evolved into semi-rhythmic generalized slow activity at 2–5 Hz, with loss of the background EEG discontinuity. The electrographic seizures did not exhibit a focal onset but appeared generalized over the scalp with amplitudes generally maximal over the anterior head region. Total seizure duration was between 30 and 90 s. The beginning of one such electrographic event is shown in Fig. 2. The inter-ictal EEG was discontinuous, with bursts of mixed frequency EEG activity interspersed by suppression. To investigate the temporal features of the DOT–EEG data, we define the electrographic onset of each event to be the average of the onsets determined by the two neurophysiologists (Table 1).

The statistical comparison between the left and right bipolar EEG channels showed that no significant hemispherical difference was present in the EEG data (*p* < 0.05).

### 4.2 Diffuse optical tomography data

**Fig. 3:**
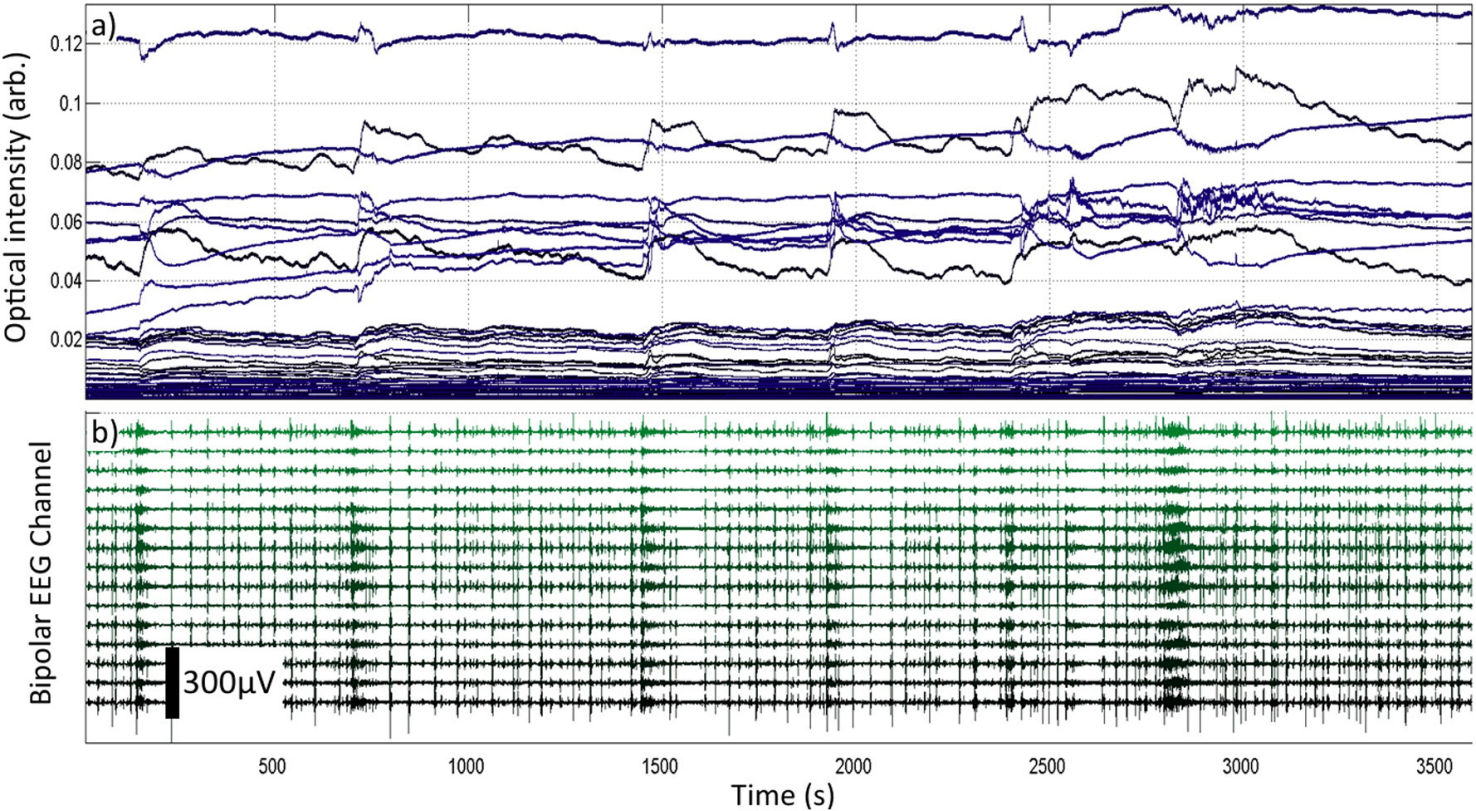
The raw DOT intensity data are shown in panel a). Panel b) shows the synchronized bipolar EEG data. Note the distinct periods of electrographic hyperactivity which are clearly visible despite the discontinuous nature of the EEG. Also note the dramatic changes in optical intensity which are temporally correlated with the EEG events.

An initial inspection of the data resulted in a total of 4 (out of 58) DOT channels being excluded due to inadequate signal quality, likely caused by poor optical coupling. The remaining set of unfiltered optical intensity measurements and bipolar EEG data are shown in Fig. 3. It is clear from the raw data that many channels exhibited features consistent with a haemodynamic response to the seizure events present in the EEG data. It also appears that there are subtle haemodynamic changes that correlate with the regular but discontinuous bursts of EEG activity between seizure events.

**Fig. 4:**
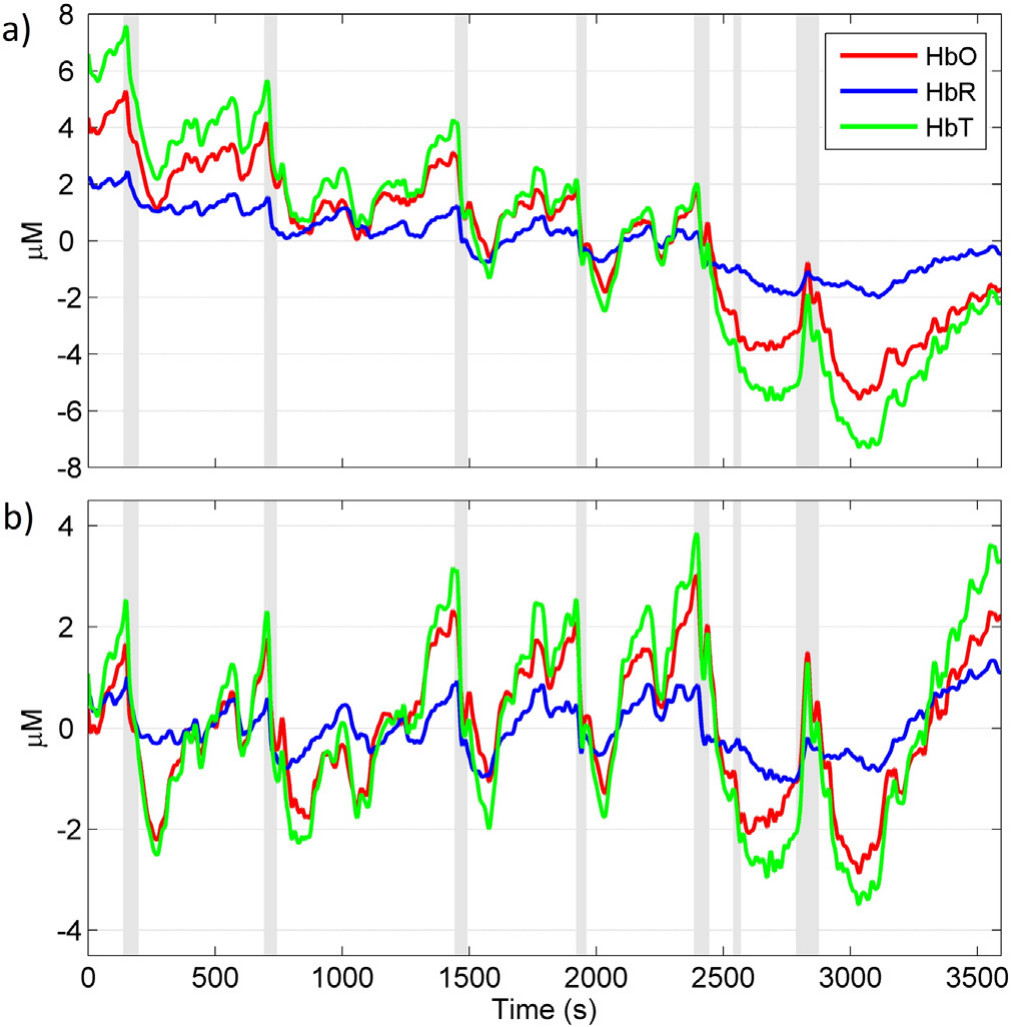
The average haemodynamic concentration changes (relative to their mean) for the hour of recording. Panel a) shows the data, high-pass filtered at 1 Hz, averaged across all good channels. The grey shaded areas show the onset and duration of the clinically identified electrographic seizures. All three HbO, HbR and HbT signals exhibit consistent behaviour across events. The events are consistently associated with an increase in HbO, HbR and HbT followed by an extended decrease below baseline. The linearly detrended version of this data is shown in panel b) to highlight the difficulty in determining the onset of the haemodynamic changes.

Inspection of the DOT data in a channel-wise manner revealed that the majority of channels exhibited a similar haemodynamic response to the seizure events. To highlight this typical response, the average changes in HbO, HbR and HbT (averaged across all channels) for the full 60 min of data are shown in Fig. 4a. Note that despite the clear linear trend, there is a remarkably consistent haemodynamic response for each event. All haemoglobin parameters show an initial increase in concentration after the onset of each event. This increase reaches a maximum at approximately 15 s after electrographic onset (on average across all channels and all 7 events). Relative to a time point 30 s prior to each seizure, the mean amplitudes (and standard deviations) of these maxima across all seizures and channels are 1.7 (2.0) µM for HbO, 1.0 (1.5) µM for HbR and 2.3 (2.9) µM for HbT. After reaching a maximum, HbO, HbR and HbT begin to decrease. This decrease continues for an average of approximately 130 s at which point the concentrations reach a minimum. After a further ~120 s, the changes in concentrations begin to recover to a steady state (see Table 1). The magnitude of the drop in haemoglobin concentrations is larger than the initial increase in most cases. The total haemodynamic response time (average duration of approximately 255 s) is longer than the duration of EEG events (Fig. 3, Table 1). Fig. 4b shows the linearly-detrended version of the data in Fig. 4a. In this data the biphasic morphology of the haemodynamic response is very clear. The detrended data also indicates that the initial increase in haemoglobin concentrations may begin prior to the electrographically defined seizure onset. In all events except event 6, the concentrations of HbO, HbR and HbT all increase prior to seizure onset.

**Fig. 5:**
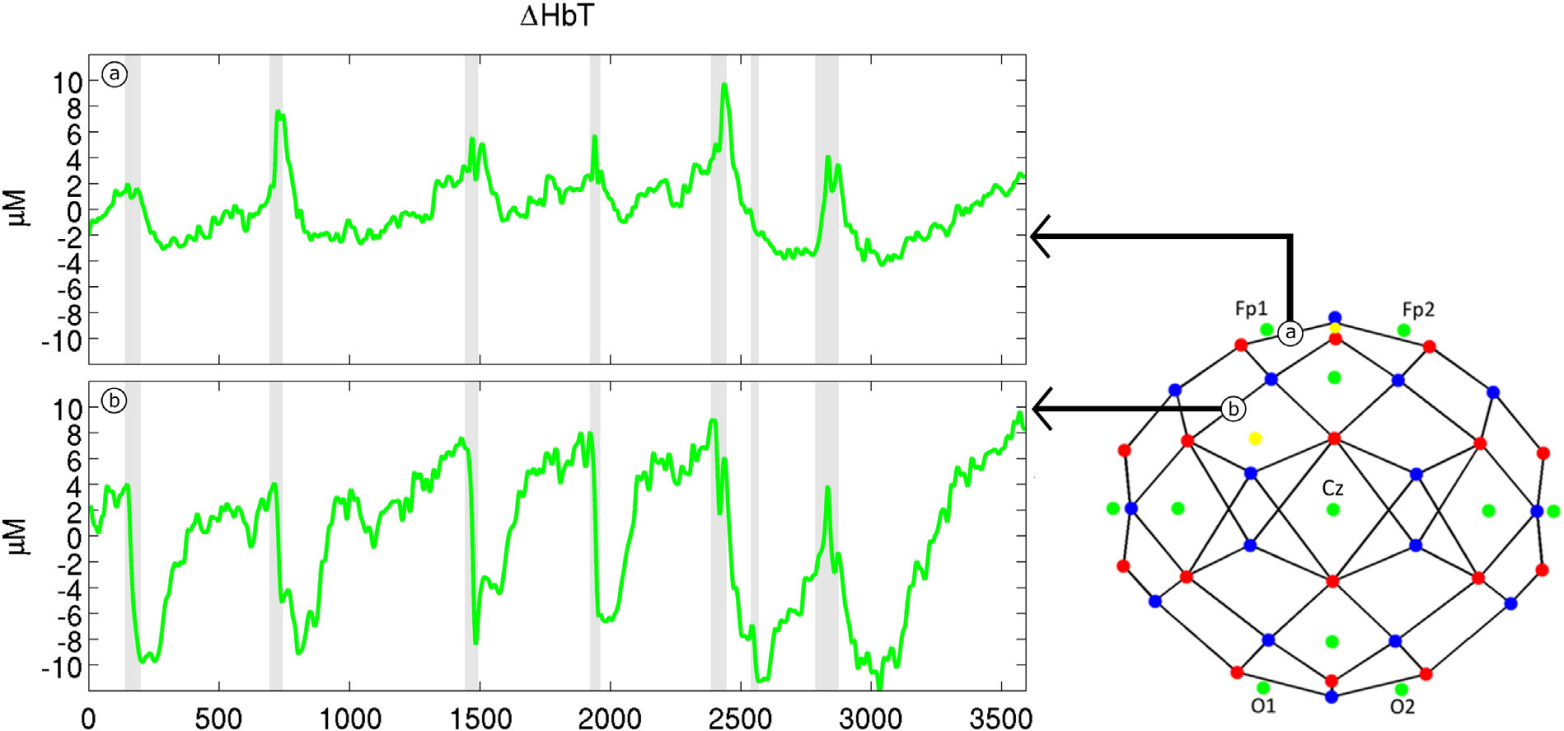
The variations in HbT for two specific channels over the left frontal lobe, relative to the mean value for the hour of acquisition. The array layout is shown to indicate the location of the two channels. The seizures are indicated by the grey shaded periods. Despite their close proximity, the channels present very different haemodynamic responses to the electrographic events. The channel shown in the upper panel (a) is dominated by increases in HbT relative to baseline while the channel shown in the lower panel (b) is dominated by decreases in HbT.

**Fig. 6:**
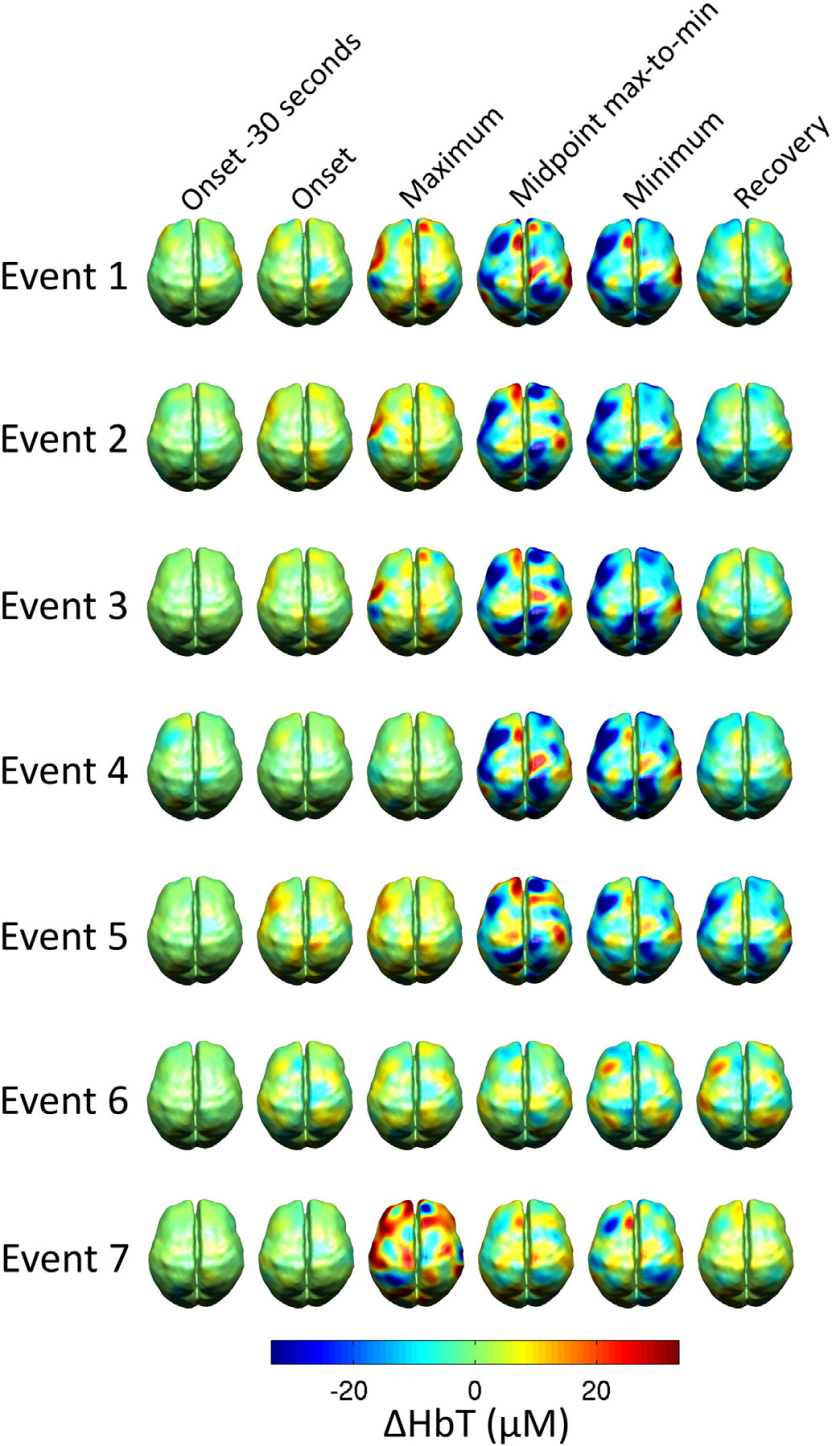
Reconstructed images of the changes in HbT associated with each of the 7 identified seizures events for 30 s prior to the electrographic onset, for the point of onset, for the time at which the spatially averaged HbT signal reaches a maximum, for the midpoint between the spatially averaged HbT maximum and the subsequent minimum, for the minimum and for the point at which the spatially averaged signal appears to have recovered to a stable state. These images are changes in HbT relative to a baseline defined as the mean of the period between 60 and 30 s prior to the electrographic seizure onset.

Although the channel-wise average presents a pronounced haemodynamic response to seizure events, the DOT data does exhibit significant spatial variation. While the majority of DOT channels exhibit the behaviour that is apparent in the spatial averages of Fig. 4, some of the channels are dominated by a prolonged increase in HbT. This variation is illustrated in Fig. 5. Two channels, both sampling the left frontal lobe, exhibit markedly different changes in HbT response to seizure events. The channel displayed in Fig. 5a is dominated by increases in HbT, while the channel in Fig. 5b exhibits the more typical response. This spatial variation is also apparent in Fig. 6, which displays a matrix of the reconstructed cortical changes in HbT, for all 7 events, at 6 specific time points relative to a baseline (defined as the mean of the period from 60 to 30 s prior to the onset of the event). Noticeably, there is a remarkable consistency of the spatial features, particularly for events 1–5. Event 6 exhibits a much less clear response, while the response of event 7 is dominated by an initial increase in HbT. Note that events 5–7 are not temporally distinct (Table 1).

**Fig. 7:**
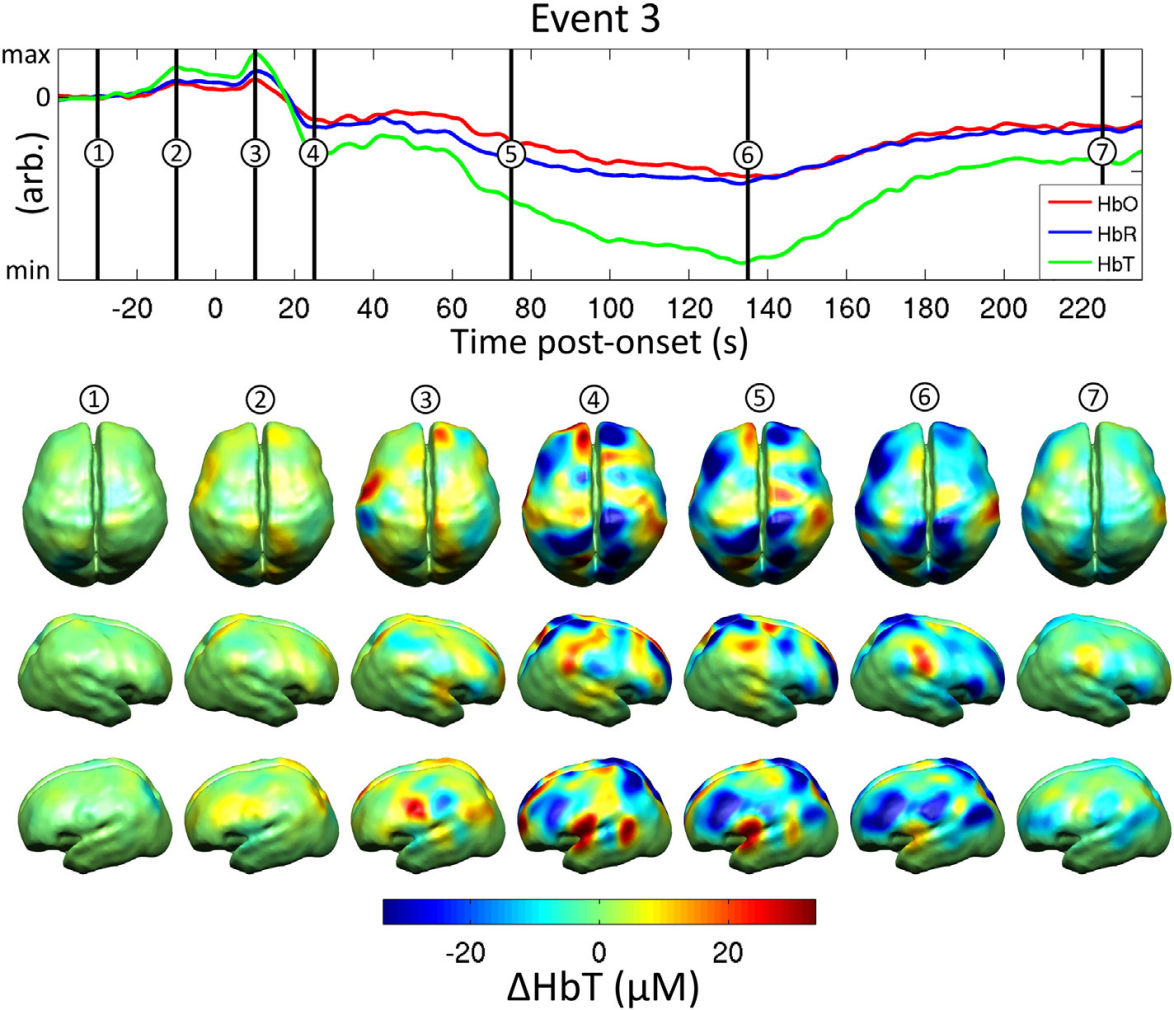
A sequence of images showing the changes in HbT associated with event 3. The upper axes show the changes in haemoglobin concentration spatially averaged across the grey matter surface. Seven distinct time-points are identified and the associate reconstructed images of the changes in HbT concentration are shown in dorsal and left and right lateral views. All data are changes relative to a baseline defined as the mean of the period between 60 and 30 s prior to the electrographic seizure onset.

Fig. 7 provides a more detailed sequence of images for event 3, and also shows the corresponding spatially averaged response. A widespread increase in HbT is apparent 10 s prior to seizure onset, with the increase most pronounced in the posterior region of the left frontal lobe. When the spatial average of HbT has reached a maximum, there are also pronounced increases in the right frontal pole and in the parietal lobes. The subsequent decrease in HbT occurs across multiple regions and is most pronounced in the left frontal lobe. Increases in HbT persist in the left fronto-temporal boundary and the right parietal lobe.

A video showing the changes in HbT and the associated synchronized EEG data for the full hour of data is available in the supplementary data associated with this paper.

## 5 Discussion

This paper presents the first DOT images of the cortical haemodynamic response to neonatal seizures. Prior investigations have been restricted to monitoring localized cerebral haemodynamics with a limited number of optode positions (Wallois et al., 2009).

Figs. 3 and 4 clearly show that distinct changes in DOT data are temporally correlated with abnormal electrographic activity. These are remarkably consistent across seizure events, predominantly consisting of an initial increase, then prolonged decrease of HbO, HbR and HbT (and therefore blood volume) for events 1–4. The haemodynamic changes associated with events 5, 6 and 7 are not temporally distinct (Table 1), but their biphasic nature is still discernable in Fig. 4. The haemodynamic response was smallest for event 6, which was considered a border-line seizure on the basis of the video-EEG.

These observed responses are very different from the standard haemodynamic response to functional stimulation observed in newborn infants (Gibson et al., 2006; Liao et al., 2010; Roche-Labarbe et al., 2014) and studies in children and adults (Lloyd-Fox et al., 2010; Obrig et al., 2000). The typical adult functional response consists of a localized increase in HbO with a smaller, co-located decrease in HbR. The magnitude of the haemodynamic changes observed here are considerably larger than the functional haemodynamic response. While the average (and standard deviation) of the seizure-induced increase in HbO across all channels and all seizures is 1.7 (2.0) µM, this value ignores the significant spatial variation exhibited across the cortex. The largest observed seizure-induced change in HbO across all seizures and channels was 12.4 µM (as calculated on a channel-wise basis without volume correction). Typical functional changes have a magnitude of 1 µM or less (Huppert et al., 2006; Gagnon et al., 2014; Lloyd-Fox et al., 2010). The extreme and prolonged decrease in blood volume that is observed in the later phase of the haemodynamic response to these events is also not consistent with any normal physiological response.

Despite the majority of the cortex exhibiting an initial increase followed by a sustained decrease in blood volume, there are cortical locations which exhibit markedly different dynamics. Areas including the left fronto-temporal and left pre-frontal cortices consistently exhibited a prolonged increase in HbT in response to electrographic seizure. An increase in blood volume and HbR has been reported at the focus of partial seizures in adults (Zhao et al., 2007), but these were accompanied by a decrease in localized HbO concentration. Despite this discrepancy, the observation of localized increases in HbT surrounded by more widespread decreases in HbT are reminiscent of the ‘centre-surround’ phenomenon apparent in animal models of focal epilepsy (Bahar et al., 2006; Suh et al., 2005; Zhao et al., 2009, 2011). It has been postulated that the physiological explanation for this phenomenon is a shunting of oxygenated blood from one region of the cortex to another in order to meet the demand of the hypermetabolic foci (Schwartz and Bonhoeffer, 2001; Zhao et al., 2011). It has also been suggested that the decreased oxygenation around the focus is related to inhibitory signalling that may have the effect of limiting the spread of the seizure (Stafstrom, 2007; Trevelyan et al., 2007). Though the mechanisms for such a process remain unclear, the widespread and dramatic decreases in HbT suggest extensive vasoconstriction across a large proportion of the cortex. The significant spatial variation illustrated in Figs. 5 and 6 highlights the importance of examining seizure haemodynamics in the imaging domain, as opposed to applying limited-channel NIRS approaches. Despite the extensive spatial variations apparent in the DOT data, the EEG presented little spatial information, and no seizure focus or hemispherical differences could be determined by either the neurophysiologists or statistical analysis.

The phenomenon of cortical spreading depression (CSD) has been associated with cerebral damage (Leao, 1944; Somjen, 2001), traumatic brain injury, migraine, stroke (Lauritzen et al., 2011) and adult seizures (Fabricius et al., 2008). It consists of a wave of cellular depolarization that has been observed to propagate at 1.7–9.2 mm/min across the cortex in stroke patients (Strong et al., 2002, 2007; Woitzik et al., 2013). This gives rise to a distinct deflection in infra-slow EEG, which can be measured by DC-coupled EEG systems (Rodin et al., 2008; Strong and Dardis, 2005). For standard EEG frequencies, CSD is associated with periods of electrical silence (Fabricius et al., 2008). Although the haemodynamic effects of CSD remains unclear in humans, animal studies suggest that large amplitude changes in cerebral haemoglobin concentrations lasting several minutes are expected (Strong and Dardis, 2005; Wolf et al., 1996). In this study we did not record DC-EEG and were therefore unable to investigate the occurrence of CSD directly. However, the common association of CSD with brain injury means the phenomena must be considered when examining pathological cerebral haemodynamics.

A number of human and animal studies have observed changes in optical parameters prior to the onset of an electrographic seizure (Zhao et al., 2007, 2011; Ma et al., 2013). There are fundamentally two possible explanations for this phenomenon: a) the recorded haemodynamic changes are a response to pathological neuronal activity that is not yet observable by scalp EEG or b) there is a true pre-ictal state that invokes distinct haemodynamic changes via some as-yet-unclear mechanism. The linear trend shown in Fig. 4a illustrates the difficulty in determining when the haemodynamic response truly begins. Furthermore, determining the onset of a seizure from a discontinuous EEG trace is very challenging and many of the seizure events are preceded by distinct bursts of activity (Fig. 2). It is therefore difficult to determine whether the seizures observed during this recording produce a haemodynamic response that begins prior to pathological EEG activity. However, it is possible to state unequivocally that in 5 out of 7 events, a dramatic increase in HbT is apparent prior to the point at which the EEG data clearly indicates a seizure (Fig. 4b).

This study represents an extension of the work presented by Cooper et al., 2011, where EEG and temporal-lobe DOT was performed in a similar cohort of brain injured newborn infants. That study presented the first evidence of large, transient, biphasic haemodynamic events in infants who were diagnosed with brain injury and seizures. However, those transients occurred in the absence of electrographic evidence of seizures or bursts were apparent in the simultaneous EEG recording. Remarkably, the scale and morphology of the haemodynamic transients are nearly identical to those presented here. This has significant implications for the use of optical imaging approaches as an addition to EEG in the investigation and detection of pathological activity in the newborn brain.

This study has a number of limitations. Perhaps most significant is the limited spatial sampling density that could be obtained with the DOT system. With a limited number of sources and detectors, a balance must be achieved between coverage and channel density. An increase in channel density with the addition of multiple short-separation (<10 mm) channels would significantly improve our ability to distinguish and remove the confounding effects of superficial haemodynamics, though these will, at least in part, be removed by the process of image reconstruction. The array design developed for this study accommodates 32 optical fibres and 13 EEG electrodes. It is a significant practical challenge to apply more sensors to an infant's head while maintaining the levels of comfort necessary to study for extended periods of time in a clinical environment. Registration of the DOT data is also a challenge. While using the patient's MRI to produce a registered atlas model was the best available approach, this process overlooked any structural brain abnormalities and also relied on the assumption that the optical fibres were located exactly at the relevant 10–5 positions. The use of a 3D digitizer to allow more accurate registration of the optical fibre positions would be a valuable addition to our acquisition process, but may hinder an already practically challenging experimental paradigm.

Practical and ethical issues preclude the routine use of conventional functional imaging technologies (including fMRI and PET) in sick newborn infants. The results of this case study, although extremely promising, do not permit general conclusions to be drawn about the cerebral haemodynamic impact of neonatal seizures. However, this study does highlight how DOT has the potential to provide clinically relevant information that is not currently available through any other mechanism. The consistency of the DOT signal observed in this infant and the possibility that abnormal haemodynamic changes precede electrographic seizure onset imply that DOT–EEG could provide improved monitoring and detection of seizures in newborn infants. Further DOT–EEG studies are already in progress with the aim of establishing the variability of these responses across seizures, infants and clinical conditions.

The ongoing advances in DOT technology are allowing continuous, high-density optical image acquisition significantly easier to achieve in challenging clinical environments. A routine prospective DOT study of infants at high risk of seizures is becoming a realistic proposition. Our group is also embarking on a series of studies using a time-resolved optical imaging system, which is expected to provide three-dimensional images of seizure haemodynamics throughout the entire infant brain. This will allow us to investigate the involvement of deeper brain structures in neonatal seizures, which is especially relevant given the electrographic data recorded using scalp EEG is unable to access these structures.

## 6 Conclusions

In this case study, we have presented the first ever DOT images of the haemodynamic response to seizures in the human infant brain. This response exhibits an abnormally large amplitude and a biphasic morphology. The spatiotemporal characteristics of this response are remarkably consistent across seizure events, implying that DOT–EEG may provide improved diagnosis and monitoring of neonatal seizures compared to EEG alone. Although more data is clearly essential, these results highlight the wealth of physiologically and clinically relevant information that can be obtained using simultaneous DOT and EEG. This study constitutes a compelling case for the development of optical imaging methods for continuous clinical application to improve seizure monitoring and our understanding of the impact of seizures on the developing brain.

The following are the supplementary data related to this article.

Supplementary Table 1 Tissue-specific optical properties

Supplementary Data: HbT_video.m4v A video showing the changes in HbT with time relative to a baseline defined as the first 30 s of recorded data. Three views of the cortical haemodynamics are provided along with the bipolar EEG data for the full hour of recording. The time point of the reconstructed images is indicated by the red vertical line superimposed over the EEG data. To produce this video the optical data was linearly detrended and down-sampled to 1 Hz. The video is sped up (30 times) such that the entire hour of data is shown in 120 s. A thumbnail for the video is shown below.

Supplementary data related to this article can be found online at http://doi.dx.org/10.1016/j.nicl.2014.06.012.

## Figures and Tables

**Table 1:** The temporal characteristics of each event determined from the video-EEG and DOT data. The EEG event durations are approximate values provided by AM. The duration of the DOT features for each event were determined on the basis of the spatially averaged (i.e. global) HbT signal (Fig. 4). The recovery times provided correspond to the approximate point at which the signal appears to return to a steady state, rather than returning to the baseline value. In the case of event 5, the latter temporal features of the haemodynamic response could not be estimated because of overlap with event 6.

Event	1	2	3	4	5	6	7
AM onset time (s)	136	692	1442	1920	2384	2539	2780
DH onset time (s)	141	694	1443	1923	2386	−	2793
Electrographic onset time (s)	138.5	693	1442.5	1921.5	2385	2539	2786.5
EEG event duration (s)	60	50	50	40	60	30	90
Time to HbT max (s)	12.5	15	10.5	3.5	14	6	46.5
Time HbT max-to-min (s)	116	128	124	111	119	128	198
Time HbT min-to-recovery (s)	123	115	112	189	−	42	142
DOT event duration (s)	251.5	258	246.5	192.5	−	172	386.5
