# Supplementary material for: Mapping cortical haemodynamics during neonatal seizures using diffuse optical tomography: A case study
Source: Neuroimage Clin. 2014 Jul 6;5:256–65. doi: 10.1016/j.nicl.2014.06.012 (PMC4141980; doi:10.1016/j.nicl.2014.06.012)
Supplement: Supplementary Table 1 — Tissue-specific optical properties [file mmc1.pdf]

Supplementary Table 1. Tissue-specific optical properties

Tissue	780 nm		850 nm	
	μ_a (mm^{-1})	μ_s' (mm^{-1})	μ_a (mm^{-1})	μ_s' (mm^{-1})
**Extra-cerebral**	0.0164	0.8376	0.0196	0.7505
**CSF**	0.0023	0.1013	0.0035	0.1660
**GM**	0.0183	0.8032	0.0192	0.6726
**WM**	0.0175	1.1548	0.0021	1.0107

Supplementary Data: HbT_video.m4v. A video showing the changes in HbT with time relative to a baseline defined as the first 30 seconds of recorded data. Three views of the cortical haemodynamics are provided along with the bipolar EEG data for the full hour of recording. The time point of the reconstructed images is indicated by the red vertical line superimposed over the EEG data. To produce this video the optical data was linearly detrended and down-sampled to 1 Hz. The video is sped up (30 times) such that the entire hour of data is shown in 120 seconds. A thumbnail for the video is shown below.
